# A Novel Family of Human Leukocyte Antigen Class II Receptors May Have Its Origin in Archaic Human Species*

**DOI:** 10.1074/jbc.M113.515767

**Published:** 2013-11-08

**Authors:** Sebastian Temme, Martin Zacharias, Jürgen Neumann, Sebastian Wohlfromm, Angelika König, Nadine Temme, Sebastian Springer, John Trowsdale, Norbert Koch

**Affiliations:** From the ‡ Section of Immunobiology, Institute of Genetics, University of Bonn, 53115 Bonn, Germany,; § Department of Molecular Cardiology, University of Düsseldorf, 40225 Düsseldorf, Germany,; ¶ Physics Department, Technical University Munich, 85747 Garching, Germany,; ‖ Biologisch-Medizinisches Forschungszentrum, University of Düsseldorf, 40225 Düsseldorf, Germany,; ** Forschungszentrum Caesar, 53175 Bonn, Germany,; ‡‡ Jacobs University, 28759 Bremen, Germany, and; §§ Division of Immunology, Department of Pathology, University of Cambridge, Cambridge CB2 1QP, United Kindgom

**Keywords:** Antigen Presentation, Major Histocompatibility Complex (MHC), Protein Assembly, Protein Domains, Protein Evolution, HLA Class II, Invariant Chain

## Abstract

HLA class II α and β chains form receptors for antigen presentation to CD4^+^ T cells. Numerous pairings of class II α and β subunits from the wide range of haplotypes and isotypes may form, but most of these combinations, in particular those produced by isotype mixing, yielded mismatched dimers. It is unclear how selection of functional receptors is achieved. At the atomic level, it is not known which interactions of class II residues regulate selection of matched αβ heterodimers and the evolutionary origin of matched isotype mixed dimer formation. In this study we investigated assembly of isotype-mixed HLA class II α and β heterodimers. Assembly and carbohydrate maturation of various HLA-class II isotype-mixed α and β subunits was dependent on the groove binding section of the invariant chain (Ii). By mutation of polymorphic DPβ sequences, we identified two motifs, Lys-69 and GGPM-(84–87), that are engaged in Ii-dependent assembly of DPβ with DRα. We identified five members of a family of DPβ chains containing Lys-69 and GGPM 84–87, which assemble with DRα. The Lys/GGPM motif is present in the DPβ sequence of the Neanderthal genome, and this ancient sequence is related to the human allele DPB1*0401. By site-directed mutagenesis, we inspected Neanderthal amino acid residues that differ from the DPB1*0401 allele and aimed to determine whether matched heterodimers are formed by assembly of DPβ mutants with DRα. Because the *0401 allele is rare in the sub-Saharan population but frequent in the European population, it may have arisen in modern humans by admixture with Neanderthals in Europe.

## Teaser

**Background:** How are isotype-mixed HLA class II heterodimers selected from the large pool of α-β subunit combinations?

**Results:** Two critical sequences in DPβ and the chaperone invariant chain control assembly of isotype-mixed HLA-DRαDPβ heterodimers.

**Conclusion:** A substantial proportion of HLA-DPβ allotypes, with the DPβ motif Lys-69/GGPM-(84–87), form matched heterodimers with DRα.

**Significance:** This DPβ motif was also found in the genome of Neanderthals, hinting at its origin.

## Introduction

Major histocompatibility class II (MHCII) molecules present antigenic peptides to T cells. In humans the majority of MHCII molecules are composed of isotype-matched α and β subunits from HLA-DR, DP, or DQ. Assembly of α and β chains, which takes place after biosynthesis in the ER,^3^ may involve the initial formation of mixed isotype complexes (1). Why isotype-matched assembly of MHCII subunits is more efficient than inter-isotype αβ pairing is still unclear. The primary sequence of MHCII α and β chains may endow properties that ensure isotype-specific interactions. In addition to isotypic differences, the polymorphism of the MHCII genes modifies the ability of the encoded α and β subunits to acquire the appropriate conformation (2, 3). A quality control of the assembled MHCII heterodimers in the ER precedes subsequent adaption of the polypeptides to the class II processing pathway. In 1992 Anderson and Miller (4) showed that invariant chain (Ii) is a chaperone for MHCII molecules that affects the conformation of the αβ heterodimer. By transfection of class II cDNAs, it was demonstrated that some MHCII allotypes assemble independent of Ii, whereas for other α and β combinations, co-expression of Ii is required for pairing and subsequent cell surface expression (5). Intracellular transport of haplotype-mixed (α and β chains encoded on different parental chromosomes) MHCII heterodimers is promoted by associated Ii chain (5). In concert with the chaperone role of Ii, Ii gene-deficient mice showed a strongly reduced expression of MHCII on the plasma membrane of their antigen-presenting cell, and again the level of surface exposure was shaped by the MHCII haplotype (6). Recent data suggest that Ii is a specialized chaperone that facilitates assembly of matched MHCII heterodimers and segregation of disfavored combinations (7). At the atomic level, it is not entirely clear which interactions of Ii with MHCII residues regulate folding of the αβ heterodimer.

As intermediates of class II assembly, the various α and β subunits form macromolecular complexes in the ER (8). Subsequent subunit assembly is a requirement for disaggregation. In principle, β subunits from any class II locus, expressed in a heterozygotic antigen-presenting cell, can interact with DRα, which is monomorphic, or with any of the two allotypes each of DPα and DQα. Because of the many combinations that are statistically possible, a random interaction of nascent α and β chains in the ER would mean that initially a substantial number of isotype-mismatched αβ pairs is formed. Dissociation of mismatched and selection of matched αβ heterodimers is required for production of functional class II peptide receptors (1). In mice and humans, respectively, a combination of certain IA with IE or of DR with DP or DQ subunits to isotype-mixed heterodimers was demonstrated in transfected fibroblast cells and in EBV-transformed B cells (9–11). Some inter-isotype paired IA/IE heterodimers were functional in mixed lymphocyte reaction and by antigen presentation assays (12, 13). In addition, mice that only expressed mixed haplotype class II molecules were able to mediate selection of functional CD4^+^ T cells (14). Structural constraints for the formation of intra- or inter-isotype pairing of MHCII heterodimers have not yet emerged.

Meanwhile, sequences of several hundred DR, DQ, and DP alleles have been identified, and the structures of the corresponding heterodimers can be devised based on the published x-ray crystal structure of MHCII heterodimers (EMBL-EBI; Refs. 15 and 16). The amino acid (aa) residues that form the contact sites between α and β chains in the polymorphic MHCII α1β1 domain are assumed to regulate intracellular assembly of the heterodimers (17). Conserved residues in the β2 domain underneath the peptide binding groove are also important for determining MHCII αβ chain pairing (18). We set out to investigate the assembly of HLA-DR/DP isotype-mixed α and β subunits with Ii.

## EXPERIMENTAL PROCEDURES

### 

#### 

##### Reagents, Cells, and Antibodies

The Ii- and MHCII-negative human lung fibroblast cell line IMRS was purchased from the Coriell Institute (Camden, NJ), and COS-7 cells were from the American Type Culture Collection. The melanoma cell line MelJuSo and mAb DA6.147 (19) were kind gifts from Dr. G. Moldenhauer (DKFZ, Heidelberg, Germany). Rabbit polyclonal antibody S35 against DRβ and mouse monoclonal anti-DR antibodies TAL1B5 (DRα), I251SB, 2.06, LGII-612.14, L243, and anti-Ii (Bu43) have been described (20–26). DA6.147 and TAL1B5 mAbs detect a cytosolic determinant on DRα (19). Anti-V5 and -His monoclonal antibodies were purchased from Invitrogen. Mouse monoclonal antibodies 12B8 and 6D4 are directed against short peptide sequences, which were used for tagging and detection of recombinant DP and DQ single chains (27).

##### DNA Constructs and Mutations

HLA-DRα and DRB1*0101 cDNAs were obtained from Dr. H. Ploegh. HLA-DQA1*0301 and HLA-DQB1*0302 were obtained from Dr. U. Grüneberg. The HLA-DQ and DP allotypes described in this paper were RT-PCR-amplified from several sources of dendritic cells. All cDNAs were PCR-cloned into the expression vector pcDNA3.1/V5His/Topo (Invitrogen) and confirmed by sequencing. Ii cDNAs Ii33, Ii33M91G, and C2GnT constructs have been described previously (27, 28).

For antibody detection of the single polypeptides, a V5His epitope was added to the C terminus of DPα, DQα, and DQβ. A 6D4 epitope was appended to the C terminus of DPβ, and a second DQβ chain was constructed with the 12B8 epitope attached to the C terminus (29).

##### Immunoprecipitation, Western Blotting, and Endoglycosidase Digestion

Cells were lysed in 0.5% Nonidet-P40 (Nonidet P-40) (Sigma) containing 10 mm Tris buffered saline (Tris, pH 7.4). Lysates were precleared by incubation with CL4B-Sepharose (Amersham Biosciences). Immunocomplexes were isolated using protein A-Sepharose, washed three times with 0.25% Nonidet P-40 in Tris, pH 7.4, separated by reducing SDS-PAGE, and for Western detection transferred to Immobilon P membrane (Millipore, Schwalbach, Germany). The membrane was blocked in PBS/Roti-Block (Roth, Karlsruhe, Germany) followed by detection of the proteins with primary antibodies diluted in blocking buffer. Bands were visualized with horseradish peroxidase-conjugated rabbit anti-mouse-IgG (Sigma), goat anti-mouse-IgM (Dianova, Hamurg, Germany), or goat anti-rabbit-IgG (Sigma) followed by enhanced chemiluminescent substrate (ECL) (Amersham Biosciences) incubation. For endoglycosidase treatment, cell lysates were digested with Endo H (2000 units) and PNGase F (1000 units) overnight at 37 °C as recommended by the supplier (New England Biolabs, Frankfurt, Germany).

##### Flow Cytometry and Fluorescence Microscopy

Transfected IMRS cells were washed twice in ice-cold PBS, 2% FCS. Mouse monoclonal antibodies were added, and cells were incubated for 30 min at 4 °C. Staining of cell surface-expressed MHCII molecules was conducted with mouse anti-HLA class II mAbs. The cells were washed 3× with PBS, 2% FCS followed by incubation with Alexa488-conjugated goat anti-mouse-IgG secondary antibody (Invitrogen) for 30 min at 4 °C. Cells were washed three times and analyzed with a BD Biosciences FACScan unit. For fluorescence microscopy, cells were seeded on cover slides fixed in 4% paraformaldehyde, permeabilized with 1% Triton X-100, blocked with Roti-Immunoblock (CarlRoth), and stained with mAb LGII-612.14 and Alexa488-anti-CD63 mAb (Clone MEM-259; ExBio, Praha, Czech Republik) followed by LGII-visualization with Alexa594 coupled anti-mouse Ig (Invitrogen).

##### Transfection of Cells

The human lung fibroblast cell line IMRS as well as COS-7 cells were transfected with the cationic polymer transfection reagent jetPEI (Q-Biogene, Heidelberg, Germany). The cells were grown to 40–60% confluence in 6-well tissue culture plates. DNA and jetPEI were diluted as recommended by the supplier (1 μg of DNA/2 μl of jetPEI), mixed, incubated for 20 min, and added to the cells. After 24 h the culture medium was removed, and fresh medium was added for another 24 h. Expression of the transfected cDNAs was examined by Western blotting of cell lysates.

##### Protein Structure Modeling

The PyMOL program (54) (contour plots at a level of +3 kT (blue) and −3 kT (red); k, Boltzmann constant; T, Temperature, 300 °K) was used.

##### Delineation of an Ancient DPB Sequence

The Neanderthal sequence reads corresponding to the DPB1* gene were obtained from the UCSC database. This library contains sequences that were obtained from the DNA of three individuals found in the Vindija cave in Croatia and termed Vi33.16, Vi33.25, Vi33.26. The genome coverage of these sequences is 54.1, 46.6, and 45.2%. Sequence reads from Vi33.16, Vi33.25, and Vi33.26 (hg19) as well as pre-aligned contigs (all-hg18, UCSC database) to the DPB1 gene were extracted and re-aligned to DPB1 (NM_002121) using CLC Workbench Version 5. A nucleotide consensus sequence from the protein-coding region was determined on the basis of the information of all three individuals. If in the first instance the pre-aligned contigs or in the second instance several sequence reads of one or more individuals covering the same region show a single nucleotide polymorphism (SNP), we assumed the SNP with a high probability and (manually corrected) exported the consensus sequence. On the basis of the nucleotide consensus sequence, we delineated a protein sequence that was aligned to *Homo sapiens* alleles DPB1*0401, 0201, 0101, and 1301 and to the chimpanzee sequence Patr-DP (Fig. 7). For assembly of a Neanderthal sequence, gaps of the Neanderthal were replaced by sequences from DPB1*0401. The sequence 15–53 from *H. sapiens* and the compared chimpanzee sequence shows three polymorphic residues of which Tyr-24 is only found in the chimpanzee sequence. DPB1*0401 was converted toward the delineated ancient sequence by several steps of PCR-based mutagenesis using appropriate primer pairs.

## RESULTS

### 

#### 

##### Isotype-mixed Class II α and β Chains Co-isolate with Ii

**FIGURE 1.**
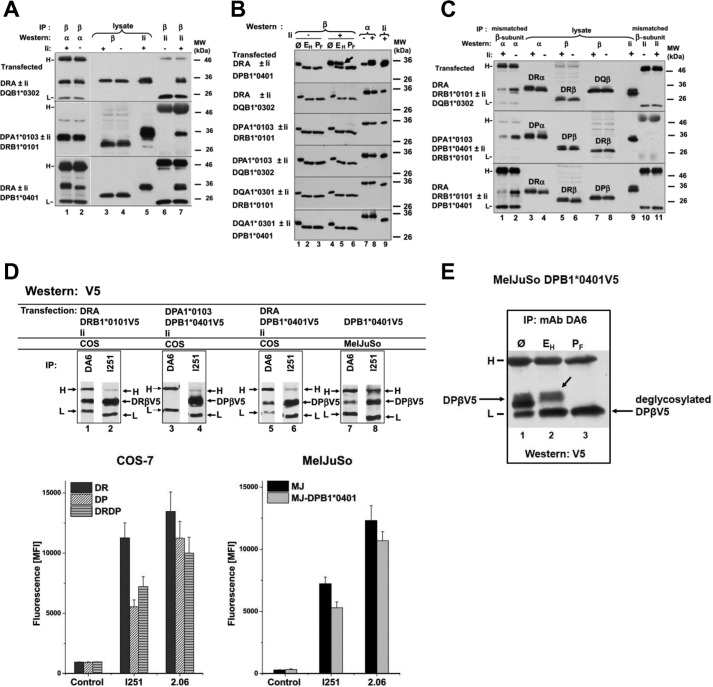
**Co-expression of isotype-mixed MHCII subunits and carbohydrate maturation in the absence or presence of Ii.** *A*, COS-7 cells were transfected with DRα (*upper* and *lower panel*)-, V5-His-tagged DQB1*0302 (*upper panel*)-, V5-tagged DPA1*0103 (*middle panel*)-, DRB1*0101 (*middle panel*)-, and 6D4-tagged DPB1*0401 (*lower panel*)-encoding cDNAs. The MHCII subunits were expressed in the presence (*lanes 1*, *3*, *5*, and *7*) or in the absence (*lanes 2*, *4*, and *6*) of Ii. Cells were lysed, and the β chains were immunoprecipitated (*IP*) by mAb V5 for V5His-tagged DQβ (*upper panel*, *lanes 1*, 2, *6*, and *7*), serum S35 for DRβ (*middle panel*, *lanes 1*, *2*, *6*, and *7*), and mAb for 6D4-tagged DPβ (*lower panel*, *lanes 1*, *2*, *6*, and *7*) and separated by SDS-PAGE. In *lanes 3–5*, cell lysates were separated. Western blotting was performed with mAb Tal1B5 against DRα (*upper* and *lower panels*, *lanes 1* and *2*), for V5-tagged DPα with V5 mAb (*lower panel*, *lanes 1* and *2*), for V5-tagged DQβ with V5 mAb (*upper panel*, *lanes 3* and *4*), for DRβ with serum S35 (*middle panel*, *lanes 3* and *4*), and for 6D4-tagged DPβ with 6D4 mAb (*lower panel*, *lanes 3* and *4*). Ii was immunoblotted in *lanes 5*, *6*, and *7* with mAb Bu43. The position of Ab-derived H and L chain bands (Abs used for immunoprecipitation) are shown on the *left*. Notice that the mobility of the Ab-epitope-tagged subunits in SDS gels decreases compared with the non-tagged polypeptides. *B*, glycosidase treatment of MHCII glycoproteins. DR, DP, and DQ α and β chains and Ii were expressed in IMRS cells as indicated. Cells were lysed, and lysates were left untreated (Ø, *lanes 1*, *4*, *7*, *8*, and *9*), treated with Endo H (*E_H_*, *lanes 2* and *5*), and with PNGase F (*P_F_*, *lanes 3* and *6*). *Lanes 1–6* were immunoblotted for the presence of β chains, *lanes 7* and *8* were immunoblotted for the presence of α chains, and *lane 9* were immunoblotted for the presence for the presence of Ii. DRα was Western-detected with mAb TAL1B5, DRβ with polyclonal rabbit Ab S35, V5-His-tagged DPα and DQα with mAb V5, and 6D4-tagged DPβ with mAb 6D4 and 12B8-tagged DQβ with mAb 12B8. The position of molecular mass markers are shown is the *right*. The *arrow* indicates that DRA/DPB1 was resistant to Endo-H digestion, consistent with movement to the medial Golgi. *C*, competition of isotype-matched with mismatched MHCII β chain for binding to α chain. The *upper panel* shows co-expression of the mixed isotypes DRα and DQB1*0302 with DRB1*0101. Mixed DPA1*0103 and DRB1*0101 with DPB1*0401 are shown in the *middle panel*, and DRα and DPB1*0401 with DRB1*0101 are shown in the *lower panel*. Ii was co-expressed as indicated on *top*. The mismatched β subunit was immunoprecipitated, SDS-PAGE-separated, and blotted for co-isolated α chain (*lanes 1* and *2*) or for Ii (*lanes 10* and *11*). Cell lysates were separated in *lanes 3–9*, and immunoblotted for the subunits indicated on *top*. The positions of the molecular mass markers are shown on the *right. D*, isolation of β-V5 class II subunits with DR-specific mAbs. COS-7 cells were transfected with Ii and with DRA.DRB1*0101 (*lanes 1* and *2*), with DPA1*0103, DPB1*0401 (*lanes 3* and *4*), with DRA.DPB1*0401 (*lanes 5* and *6*) and immunoprecipitated with mAbs DA6.147 or I251SB. In *lanes 7* and *8* MelJuSo cells stably transfected with DPB1*0401-V5 were lysed and immunoprecipitated with mAb DA6.147 or I251SB (*lanes 7* and *8*). *Lanes 1–8* were Western-blotted with V5 mAb. *Lower panel*, transfected cells were stained with mAbs I251 and 2.06 to demonstrate surface expression of DRαβ, DPαβ, or DRDPB1*0401 molecules by flow cytometry. *Control* indicates background staining of an Ig isotype control. MelJuSo cells stably transfected with DPB1*0401 (MJ-DPB1*0401) were compared with MelJuSo cells (*MJ*). *E*, mAb DA6.147 (against DRα) was used for immunoprecipitation of cell lysates from MelJuSo cells stably transfected with DPB1*0401-V5. Lysates were digested with Endo H (*E_H_*, *lane 2*), with PNGase F (*P_F_*, *lane 3*), or left untreated (Ø, *lane 1*). SDS-PAGE gels were blotted, and specific bands were visualized with V5 mAb. *Arrow*, *lane 2* indicates Endo H-resistant DPB1*0401-V5 band. *D* and *E*, *arrows* label the position of DRβ-V5, of DPβ-V5, and of H and L bands derived from Abs used for immunoprecipitation.

To explore potential inter-isotype pairing of MHCII subunits, three combinations of α and β chains, DRαDQβ, DPαDRβ, and DRαDPβ, were expressed in the presence or absence of Ii in COS-7 cells. Subsequently, cells were lysed, and MHCII molecules were immunoprecipitated with a mAb against the V5-tagged DQβ chain, with a serum against DRβ, and with mAb against the 6D4-tagged DPβ chain. The co-isolated immunoprecipitates were SDS-PAGE-separated and immunoblotted for co-expressed α chains (Fig. 1*A*, *lanes 1* and *2*), or for Ii (*lanes 6* and *7*). The α chain was detected in β chain immunoprecipitates of all three αβ combinations. Co-isolation of α chain was independent of the presence of Ii, although Ii was found in all complexes when it was expressed, as demonstrated in *lane 7*. Expression of β chain and of Ii was confirmed by immunoblotting of cell lysates (*lanes 3–5*). The result indicates that α and β chains of different isotypes co-isolate and that these complexes contain Ii.

##### Carbohydrate Maturation of DRA.DPB1*0401 Mixed Isotype Molecules Indicates That They Are Transported from the ER to the Medial Golgi

Unassembled α or β chains (intermediates formed after biosynthesis) or aberrantly folded complexes of MHCII subunits do not usually exit the ER (30). In contrast, properly folded MHCII heterodimers are transported with Ii from the ER to a medial Golgi compartment, where the glycoproteins acquire modification of their *N*-linked oligosaccharides. Carbohydrate maturation of the MHCII subunits was studied in the following experiments in the Ii and class II-negative human lung fibroblast cell line IMRS. Several combinations of DR, DP, and DQ subunits were expressed in IMRS cells. Cells were lysed, and lysates were digested with Endo H or with PNGase F (PNGase F completely cleaves off the *N*-linked carbohydrates of both complex and of high mannose type glycans, whereas Endo H only cleaves the high mannose type), SDS-PAGE-separated, and subsequently immunoblotted for β chain (Fig. 1*B*). Six α and β subunit combinations are displayed in Fig. 1*B*. A mobility shift of the class II β chain of untreated (Fig. 1*B*, *lane 1*) compared with Endo H-treated cell lysates (Fig. 1*B*, *lane 2*) indicated cleavage of the high mannose carbohydrate. For all examined α and β combinations, the position of the Endo H-sensitive β chain was comparable to the mobility of the β chain digested with PNGase F (Fig. 1*B*, *lane 3*). An effect of Ii on carbohydrate processing had been demonstrated by transfection of DR α and β and inclusion of Ii cDNA (31); hence, we examined the impact of Ii on oligosaccharide maturation of the isotype-mixed α and β glycoproteins. In the presence of Ii (Fig. 1*B*, *lanes 4–6*), one of six examined combinations, namely DRA.DPB1*0401, underwent no change of mobility of DPβ (Fig. 1*B*, *upper panel*, *lane 5*, arrow), indicating resistance of the *N*-linked glycan to treatment with Endo H. This difference is due to carbohydrate processing upon delivery to the medial Golgi. The other examined αβ combinations remained Endo H-sensitive, presumably because they were retained in the ER. Expression of the polypeptides is shown by Western blotting of cell lysates (Fig. 1*B*, *lanes 7–9*). This experiment indicates export of DRA.DPB1*0401, but not other mixed isotype molecules, from the ER to the medial Golgi.

##### Competition of Isotype-matched with -mismatched β Chains for Binding to the α Subunit

We next investigated whether an isotype-matched β chain can compete with the isotype-mismatched β chain for binding to α, which could explain the dominant presence of isotype-matched αβ heterodimers in antigen-presenting cells (Fig. 1*C*). IMRS cells were transfected as in Fig. 1*A* but with addition of the isotype-matched β chain. Cells were lysed, and the isotype-mismatched β-chain was immunoprecipitated and Western-blotted for the presence of associated α chain (Fig. 1*C*, *lanes 1* and *2*). In the absence of Ii, the α chain was co-isolated with the isotypic β chain (Fig. 1*C*, *lane 2*). However, in the presence of Ii, impaired co-isolation of α subunit was apparent (Fig. 1*C*, *lane 1*). *Lane 11* demonstrates that in the presence of the isotype-matched β chain, the mismatched β chain was not associated with Ii. This competition by the isotypic β chain for its matched α chain was dependent on Ii, indicating that the isotype-matched β chain displaces the mismatched β chain in the αβIi complex. These data corroborated previous studies on competition of murine IA and IE subunits in transfected cells (32). Our data show that efficient intra-isotype HLA class II pairing is facilitated by Ii.

We wondered whether despite the presence of DRβ chain, a combination of DPB1*0401 with an isotype-mismatched α chain can be identified in antigen-presenting cells. For detection of DRαDPβ, we employed the mAb DA6.147, which detects DRα. The specificity of the mAb was verified by immunoprecipitation (Fig. 1*D*). This mAb reacted to DRαDRβ (Fig. 1*D*, *lane 1*) but not to DPαDPβ (Fig. 1*D*, *lane 3*) heterodimers, which were co-expressed with Ii in COS-7 cells. Importantly, mAb DA6.147 immunoprecipitated DPB1*0401-V5, which was co-transfected with DRα and Ii (Fig. 1*D*, *lane 5*). To explore association of DPB1*0401-V5 with endogenous α chains, the MHCII-positive human melanoma cell line MelJuSo was stably transfected with V5-tagged DPB1*0401 encoding cDNA. Immunoprecipitation of cell lysates from MelJuSo-DPβV5 with mAb DA6.147 showed that DPβ-V5 was isolated (Fig. 1*D*, *lane 7*). In principle, DPαDPβ and DRαDRβ could be co-isolated if these two class II isotypes bound to the same Ii trimer, which would explain detection of DPβ-V5. However, this possibility can be ruled out as we showed recently that only one class II α chain can be detected in class II/Ii oligomers (8). Because mAb DA6.147 does not react with DPαDPβ, this result suggests that DPβ-V5 was co-isolated with isotype-mismatched endogenous α chain, presumably with DRα. For comparison, mAb I251SB was used, which reacts with DR and cross-reacts with DP molecules (Fig. 1*D*, *lanes 2*, *4*, *6*, and *8*). The amount of DPβ-V5 immunoprecipitated by mAb I251SB exceeded immunoisolation by mAb DA6.147, which in part can be explained by the preference of DPβ-V5 to associate with endogenous DPα rather than DRα. FACS profiles of class II stained transfected cells are shown in Fig. 1*D*, *lower panel*.

To demonstrate carbohydrate maturation of DPB1*0401, which was co-isolated with DRα, we digested DA6.147 immunoprecipitates with Endo H or with PNGase F and blotted for DPB1*0401-V5 (Fig. 1*E*). The Endo H-treated immunoprecipitate (Fig. 1*E*, *lane 2*) shows a glycosidase resistant DPB1*0401 band (Fig. 1*E*, *lane 2, arrow*) co-migrating with the undigested immunoprecipitate (Fig. 1*E*, *lane 1*). Deglycosylated DPB1*0401-V5 runs in the SDS gel in position of immunoglobulin light chain (Fig. 1*E*, *lane 3*, *L*).

##### DRA.DPB1*0401 Isotype-mixed Dimers Bind Peptide

**FIGURE 2.**
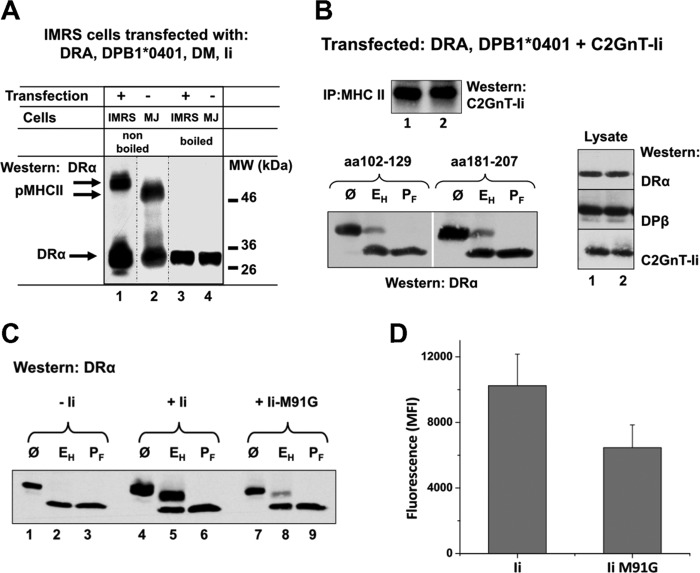
Inspection of the peptide binding groove of DRA.DPB1*0401. *A*, detection of SDS-resistant bands in DRα- and DPβ-, DM-, and Ii-transfected cell lysates. Transfected IMRS cells and MelJuSo (*MJ*) cells were lysed, and lysates were incubated for 1 h at room temperature (non-boiled, *lanes 1* and *2*) or boiled (*lanes 3* and *4*), separated by SDS-PAGE, and immunoblotted for DRα with mAb TAL1B5. The SDS-resistant band representing peptide-loaded MHCII heterodimers is labeled with pMHCII. The position of single DRα chain is indicated on the *left. M*_r_ markers are shown on the *right. B*, co-expression of DRA.DPB1*0401 with C2GnT-Ii Fusion proteins. IMRS cells were transfected with DRA.DPB1*0401 and with C2TnG-Ii (*1*) and (*2*) constructs. In construct 1, the CLIP sequence was replaced by the C2GnT sequence aa 102–129 and in construct 2 by the C2GnT sequence aa 181–207. Immunoprecipitation (*IP*) of DRA.DPB1*0401 by DR mAb and Western blotting for V5-tagged C2TnG-Ii fusion protein is shown in the *upper panel. Lower panel*, cell lysates were digested with Endo H (*E_H_*), with PNGase F (*P_F_*), or left untreated (Ø) and subsequently SDS-PAGE separated and Western-blotted for DRα. Expression of the transfected cDNAs is shown in *lanes 1* and *2. C*, mutant Ii impacts on DRα glycosylation. IMRS cells were transfected with cDNAs encoding DRα and DPB1*0401 in the absence of Ii (*lanes 1–3*), in the presence of Ii (*lanes 4–6*), or in the presence of Ii M91G mutant (Ii M91G). Cells were lysed, and lysates were digested with Endo H (*E_H_*) (*lanes 12*, *5*, and *8*) and with PNGase F (*P_F_*) (*lanes 3*, *6*, and *9*) or left untreated (Ø) (*lanes 1*, *4*, and *7*). Lysates were separated by SDS-PAGE and Western-blotted with mAb TAL1B5 for the presence of DRα. *D*, transfected cells as in *C* were stained with mAb 2.06 and analyzed by flow cytometry. Mean fluorescence intensity (*MFI*) is shown. S.D. is based on five individual experiments.

We next examined whether DRA.DPB1*0401 heterodimers can bind peptides by inspection for SDS resistance of MHCII molecules at room temperature. To acquire a peptide-receptive conformation, MHCII heterodimers require the presence of HLA-DM (DM), which interacts with MHCII and catalyzes exchange of a fragment of Ii for endosomal peptides (33, 34). IMRS cells were transfected with Ii-, DM-, and DRα- and DPB1*0401-encoding cDNAs. Subsequently, cells were lysed, and lysates from transfected cells and, for comparison, lysates from MelJuSo cells, were subjected to SDS-PAGE. Fig. 2*A* shows non-boiled (*lanes 1* and *2*) and boiled (*lanes 3* and *4*) samples, which were Western-blotted for DRα. In non-boiled samples, two bands were apparent (Fig. 2*A*, *lane 1*), one at ∼60 kDa and one in the position of single DRα chain. The band with the lower mobility in SDS gels represents compact dimers. For comparison, peptide-loaded DR αβ heterodimers from MelJuSo cells are shown in Fig. 2*A*, *lane 2*. The αβ/peptide complexes dissociated by thermal denaturation, yielding free α chains (Fig. 2*A*, *lanes 3* and *4*). The result in Fig. 2*A* indicates that, in the presence of DM, the DRA.DPB1*0401 heterodimer was recovered as a heat-resistant band, consistent with intracellular binding of peptide.

##### The Groove Binding Sequence of Ii Confers a Conformation of DRA.DPB1*0401 Heterodimer Susceptible to Carbohydrate Maturation

Proper assembly of DRα with DPB1*0401 depends on the presence of Ii, possibly the groove binding segment of the Ii protein. To investigate this, we employed recombinant Ii chains in which the groove binding sequence was replaced by the pancreatic C2GnT antigen (28). We examined two C2GnT-Ii fusion proteins that differ by their antigenic sequences for co-isolation with DRA.DPB1*0401 from transfected cells (Fig. 2*B*, *lanes 1* and *2*, *upper panel*). Immunoblotting of Ii showed that both C2GnT-Ii constructs were co-isolated with DRA.DPB1*0401 immunoprecipitates. Subsequently, we asked whether C2GnT-Ii can substitute for native Ii in controlling normal glycosylation of DRα in the DRA.DPB1*0401 complex. Digestion of cell lysates with Endo H demonstrated that in both C2GnT-Ii-transfected cell batches only minimal amounts of DRα became resistant to Endo H (Fig. 2*B*, *lower panel*). Together, the results in Figs. 1*B* and 2*B* suggest that interaction of native Ii to DRA.DPB1*0401 imparts a conformational change that is required for a normal glycosylation of DRα. The C2GnT sequence was not able to substitute in this function.

By mutation of DPβ, it was previously demonstrated that occupation of the P1 pocket in the peptide binding groove is sufficient to partially stabilize the class II heterodimer (35). To explore the role of P1 for assembly of DRα with DPB1*0401, we employed the mutant Ii M91G. In this mutant, an anchor residue of Ii, important for binding of Ii to the pocket P1 of DRαβ, was altered. Ii M91G still binds to DRαβ but exhibits a decreased interaction with DRα (27). Co-expression of Ii M91G with DRA.DPB1*0401 is shown in Fig. 2*C*. IMRS cells were transfected with DRA.DPB1*0401 in the absence of Ii (Fig. 2*C*, *lanes 1–3*) in the presence of Ii (Fig. 2*C*, *lanes 4–6*), or in the presence of Ii M91G (Fig. 2*C*, *lanes 7–9*). Cell lysates were digested with glycosidases, SDS-PAGE-separated, and Western-blotted for DRα. In the presence of Ii M91G, only a small portion of DRα acquired Endo H resistance (Fig. 2*C*, *lane 8*, *upper band*), which was monitored as a band with increased mobility compared with undigested DRα (*lane 7*). Most of the DRα chain in *lane 8* was sensitive to Endo H digestion, which is displayed as a band with the mobility of PNGase F-treated DRα (Fig. 2*C*, *lane 9*). For comparison, the DRα subunit in DRA.DPB1*0401 remained Endo H-sensitive in the absence of Ii (Fig. 2*C*, *lane 2*) but became partially Endo H resistant when Ii was present, because only one of the two *N*-linked glycans of DRα became resistant to Endo H (36) (*lane 5*). This result suggests that interaction of Ii with the first pocket of DRA.DPB1*0401 has a pivotal role for assembly of the heterodimer and subsequent trimming of the *N*-linked carbohydrates in the medial Golgi. Relative cell surface expression of the MHCII heterodimers at steady state in transfected IMRS cells is shown in Fig. 2*D* (as detected by mAb 2.06). In this experiment DRΑ.DPB1*0401, when co-expressed with Ii M91G, showed only an ∼60% of surface expression of the DRΑ.DPB1*0401 co-expressed with wild type Ii. A single aa residue change in Ii thus leads to reduced carbohydrate modification and impaired surface expression of the DRα glycoprotein.

##### Polymorphic Sequences of DPβ Chains Control Carbohydrate Maturation and Surface Expression of DRαDPβ Heterodimers

**FIGURE 3.**
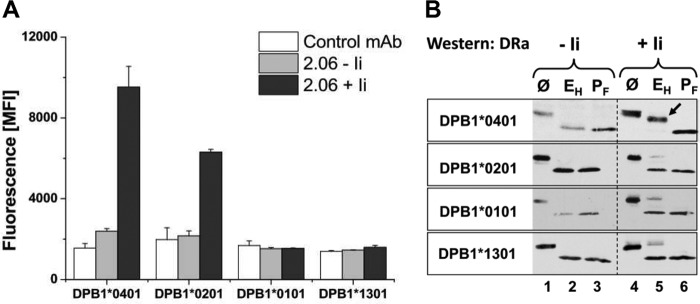
**Cell surface expression and *N*-glycosylation of four DPB1 allotypes.** *A*, variable surface expression of DPB1* allotypes co-expressed with DRα. IMRS cells were transfected with four DPB1* allotypes in combination with DRα cDNA in the presence or absence of Ii. Surface expression of MHCII was determined by flow cytometry. Mean fluorescence intensity (*MFI*) is shown. S.D. is based on three independent experiments. *B*, carbohydrate maturation of DRα chains combined with DPB1* allotypes. Four DPB1* allotypes, indicated on the *left*, were co-expressed with DRα in the absence (*lanes 1–3*) or presence of Ii (*lanes 4–6*). Cells were lysed, and lysates were left untreated (Ø, *lanes 1* and *4*), digested with Endo H (*E_H_*, *lanes 2* and *5*), or treated with PNGase F (*P_F_*, *lanes 3* and *6*), SDS-PAGE-separated, and Western-blotted for DRα with mAb TAL1B5. The *arrow* in *lane 5* (*upper panel*) indicates increased mobility of Endo H-treated DRα compared with untreated DRα bands (*lane 4*). The *lower three panels* show untrimmed glycans or limited aberrant glycosylation of DRα (*lane 5*).

The experiments above showed that the groove binding segment of Ii and, in particular, interaction of Ii with the first pocket of MHCII had a profound effect on carbohydrate trimming of DRA.DPB1*0401. Binding of Ii may confer a conformation of DRA.DPB1*0401 that is accessible to high mannose-trimming enzymes. We, therefore, investigated what role the polymorphic residues of DPβ, which are clustered in the β1 domain, have in this effect of Ii. We expressed DPB1*0401 and three other DPβ allotypes in combination with DRα in the presence or absence of Ii to see whether MHCII molecules could be detected on the surface of transfected cells. The cells were stained with 2.06, an anti DR mAb that cross-reacts with DPβ chains, and measured by flow cytometry (Fig. 3*A*). In the absence of Ii, a combination of DRα with DPB1*0401 resulted in low surface expression of the MHCII heterodimer. The membrane exposure of DRA.DPB1*0401 was strongly increased when Ii was co-expressed, confirming that Ii facilitates MHCII folding and transport. A similar result was obtained for the heterodimer DPB1*0201 and DRα, which also shows high surface expression when co-expressed with Ii. In contrast, two additional DPB1 allotypes (0101 and 1301) that were co-expressed with DRα were not detected on the cell surface of transfected cells either in the presence or in the absence of Ii. Examination of surface expression of DRα with DPβ allotypes revealed that surface detection of DPB1*0401 and 0201 depends on the presence of Ii.

We next examined the Endo H resistance of the DRα chain when co-expressed with each of four different DPB1 allotypes (Fig. 3*B*). Cells were lysed and lysates were left untreated (Fig. 3, *lanes 1* and *4*), treated with Endo H (*lanes 2* and *5*), or treated with PNGase F (*lanes 3* and *6*) and Western-blotted for DRα. In the absence of Ii, all examined DPB1* allotypes yielded a completely Endo H-sensitive DRα chain (Fig. 3, *lane 2*). In the presence of Ii, the DRα chain co-expressed with DPB1*0401 became partially Endo H-resistant (Fig. 3*B*, *lane 5*). In contrast to DPB1*0401, the DPB1*0101 and 1301 chains mostly yield Endo H-sensitive DRα despite the presence of Ii (mostly *lane 5*), suggesting that these combinations cannot travel to the Golgi apparatus and the cell surface. It is interesting that a portion of the heterodimers of DRα with DPB1*0201 acquired the ability to reach the cell surface (Fig. 3*A*) but failed to attain the conformation of associated DRα that is required for trimming of the *N*-linked glycans on DRα. A limited amount of the DRα band co-expressed with each of three DPB1* allotypes (0201, 0101, 1301) remained upon Endo H treatment in the position of undigested DRα (Fig. 3*B*). This band indicates complete resistance of the glycans to Endo H treatment, implying that both *N*-linked glycans are modified to a complex type carbohydrate. Our interpretation of the results in Fig. 3*B* is that Ii impacts on the conformation of DRA.DPB1*0401 heterodimers by limiting the extent of conformational variation and allowing normal trimming of the DRα oligosaccharides. The inappropriate conformation of the other DRαDPβ combinations results in sensitivity of both glycans to Endo H digestion and in aberrant carbohydrate maturation of small amounts of DRα. Our results suggest that in addition to Ii-induced conformational changes, polymorphic residues of DPβ are important for conversion of the class II heterodimer to the final conformation.

##### Which Polymorphic DPβ aa Residues Influence Glycosylation of DRα?

**FIGURE 4.**
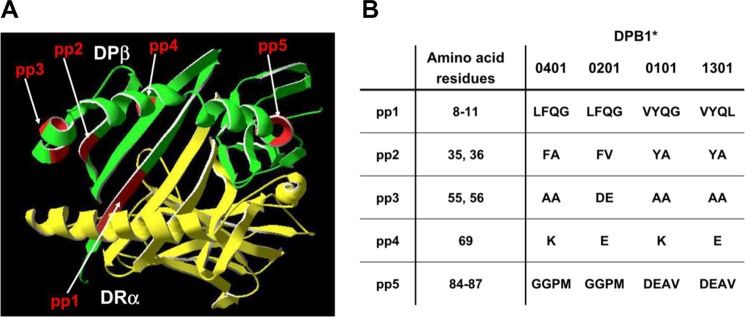
**Polymorphic sequences on DPB1 allotypes.** *A*, three-dimensional structure of α1β1 domain of DRαDPβ. The structure of DRα (*yellow*) was drawn from DRαDRβ, and DPβ (*green*) was drawn from DPαDPβ images and combined to the isotype-mixed DRαDPβ heterodimer. Polymorphic aa residues were cumulated in polymorphic patches pp1 to pp5 (*red*). *B*, polymorphic residues of the β1 domain of DPB1* allotypes. Four DPB1* allotypes were compared in their polymorphic patches pp1 to pp5.

We next investigated which aa residues of the allotypic DPβ chains determine the initial assembly with DRα and Ii. To depict the localization of the polymorphic residues of DPβ in the DRαDPβ heterodimer, we modeled a three-dimensional structure derived from the individual DRα and DPβ structures that were obtained from the three-dimensional structure of DRαβ and DPαβ heterodimers (16, 37). The novel peptide binding groove of the mixed DRαDPβ is presented in Fig. 4*A*. Five polymorphic patches (pp1–5) of DPB1 allotypes contained in the DRαDPβ α1β1 domain are pointed out in the figure. Based on the inability of three DPB1* allotypes to form isotype-mixed dimers with DRα, we investigated further the polymorphic differences in DPβ affecting interaction to DRα and to Ii. The pp1 to pp5 allotypic aa residues of the four investigated DPΒ1 allotypes are listed in Fig. 4*B*.

**FIGURE 5.**
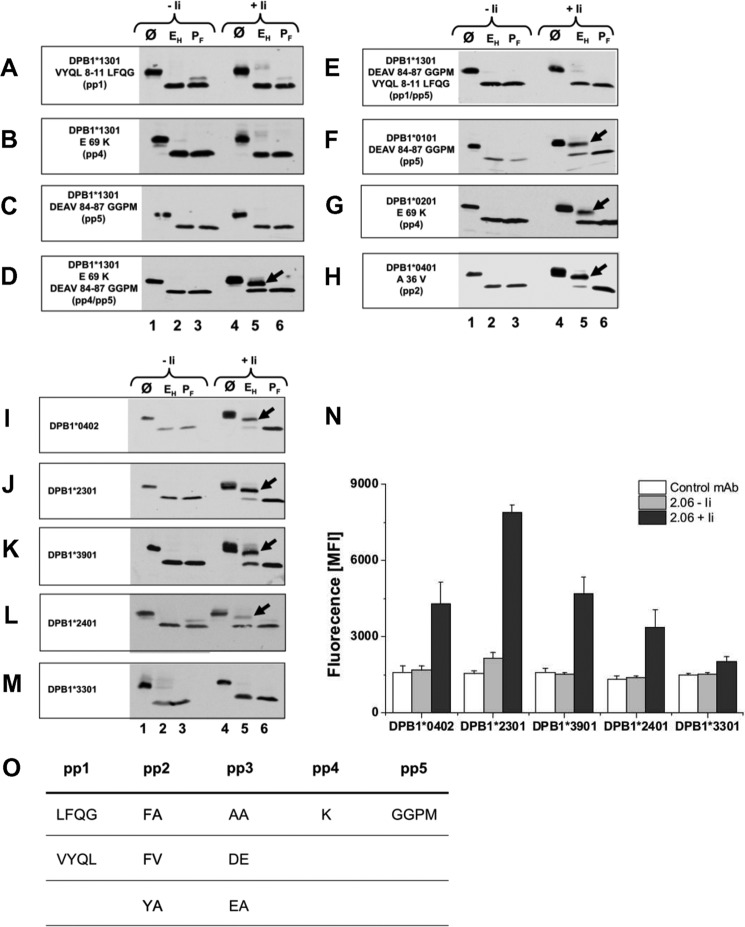
**Impact of polymorphic residues of DPβ on carbohydrate maturation and cell surface expression of isotype mixed DRαDPβ heterodimers.** *A–M*, glycosidase treatment of DRα chain co-expressed with DPβ mutants. Cells were transfected with DRα with the indicated DPβ mutants and with or without Ii. Subsequently, cells were lysed, and lysates were digested with Endo H (*E_H_*), with PNGase F (*P_F_*), or left undigested (Ø) and subjected to SDS-PAGE with subsequent Western blotting for DRα (mAb TAL1B5). *Arrows* in Endo H-digested lanes indicate the DRα chain with one Endo H cleaved and one Endo H-resistant glycan chain. *N*, cell surface detection of DRαDPβ heterodimers by cytofluorometry. Cells were transfected with DRα, DPB1* allotypes, and Ii-encoding cDNAs. DRA.DPB1* expression was monitored by staining cells with mAb 2.06. S.D. is based on four experiments. *O*, consensus sequence of five polymorphic patches from DPB1* allotypes for association with DPα.

In the following, our strategy was to introduce mutations into the DPB1*1301, 0101, and 0201 allotypes to sequentially convert aa residues of patches pp1 to pp5 toward sequences in DPB1*0401. In each experiment, the properties of the DPβ mutants were evaluated by testing Endo H resistance of the associated DRα chain co-expressed with the respective DPβ mutants. Fig. 5 (*A–C*) shows that when individual polymorphic patches of DPB1*1301 were mutated toward the DPB1*0401 sequence, carbohydrate trimming of the DRα oligosaccharides co-expressed with DPB1*1301 mutants was consistently absent even in the presence of Ii (Fig. 5, *lanes 2* and *5*). Therefore, subsequently, combinations of two polymorphic patches were mutated in DPB1*1301. These are pp4/pp5 or pp1/pp5 (Fig. 5, *D* and *E*). This time, in the presence of Ii, the DPB1*1301 (*D*) mutant pp4/pp5 yielded DRα with Endo H resistance of one *N*-linked glycan, whereas both DRα glycans in the heterodimer formed with the DPβ pp1/pp5 mutant remain Endo H sensitive (*E*, *lane 5*). Because neither mutation of pp4 or pp5 alone re-established carbohydrate maturation, this result indicates that a combination of polymorphic sequences in pp4 and pp5 of DPβ is required for normal glycosylation of associated DRα. We further investigated the role of pp4 and pp5 by mutating DPB1*0101, which has a lysine residue as pp4 but differs in pp5 by DEAV (aa 84–87) from allotype 0401 (Fig. 5*F*, *lane 5*, *arrow*). A mutation of pp5 to GGPM in DPB1*0101 yielded, in concert with the above results, normal glycosylated DRα in the presence of Ii.

In Fig. 5, *G* and *H*, the impact of pp4 and pp2 mutations is shown with the modified DPB1* allotypes 0201 and 0401. The Glu-69 residue in 0201 was mutated to Lys and Ala-36 was mutated in 0401 to a Val residue. Both DPβ mutants yielded Endo H resistance of DRα in the presence of Ii.

Taken together, the results in Fig. 5, *A* to *H*, strongly support the conclusion that a combination of Lys-69 and GGPM-(84–87) in pp4, and pp5 is critical for appropriate interaction of DRα with DPβ and Ii, whereas the allelic sequences in pp1, pp2, and pp3 have no substantial impact on Endo H resistance of DRα. Intriguingly, an inspection of DPβ GenBank^TM^ sequences revealed that ∼20% of the presently known DPβ alleles encode both Lys-69 and GGPM-(84–87) (not shown). Therefore, many DPβ allotypes could potentially form mixed pairs with DRα.

##### A Family of DPB1 Allotypes Forms Isotype-mixed Heterodimers with DRα

To confirm the importance of pp4 and pp5 for association of DPβ with DRα, we examined the efficacy of naturally occurring DPβ allotypes to mediate carbohydrate trimming of DRα glycan. We generated four additional DPβ allotypes containing Lys-69 and GGPM-(84–87) by mutating sequences of related allotypes and tested them for interaction to DRα. By mutation of pp4 in DPB1*0201 and L178M in the β2 domain, we generated the allotype 0402 (Fig. 5*I*). This mutant, which contains pp4 and pp5 of the DPB1*0401 allotype but differs in A36V (pp2) and AA55,56DE (pp3), was co-expressed with DRα and Ii. We inspected whether or not their *N*-linked DRα carbohydrates had been modified in the Golgi. *N*-Glycanase digestion demonstrated partial Endo H resistance of the DRα oligosaccharides (Fig. 5*I*, *lane 5*, *arrow*). This result indicates that in addition to 0401, the 0402 allele of DPB1* also facilitates glycosylation of DRα. Mutation of DPB1*0401 to A36V, F35Y, and A55E yielded the alleles DPB1*2301, 2401, and 3901. The mutated chains were co-expressed with DRα and Ii. In the presence, but not in the absence of Ii, the DRα chain co-expressed with each of the three DPβ allotypes yielded partial Endo H resistance (Fig. 5, *K–L*, *lanes 5*, *arrows*), providing evidence for the existence of a family of DPβ allotypes that associate with DRα and mediate normal carbohydrate trimming of one DRα bound glycan. As with all allotypes tested, the proper glycosylation pattern of DRα depended on the presence of Ii.

To demonstrate that aberration from this consensus sequence abrogates proper glycosylation of DRα, we generated the allele 3301 by substituting lysine 69 in DPB1*0401 for glutamate. According to our previous results, this mutant should yield no trimming of the associated DRα glycoprotein. As shown on Fig. 5*M*, glycosidase treatment of the DPB1*3301-associated DRα did not lead to Endo H resistance of one *N*-linked glycan. The process of DRα carbohydrate maturation failed, and the glycans remained the high mannose type.

Subsequently, surface expression of the five combinations of DRα with DPB1* allotypes was inspected in the presence or absence of Ii by flow cytometry (Fig. 5*N*). Staining of transfected cells with mAb 2.06 revealed that DPB1* chains 0402, 2301, 3901, and 2401 yielded substantial surface expression in the presence of Ii but not without Ii, whereas only minor amounts of DRA.DPB1*3301 were detected on the cell membrane. This result is consistent with detection of Endo H resistant DRα chains in Fig. 5, *I* to *M*. A consensus sequence for the compared DPΒ1* mutants and allotypes, which form mature heterodimers with DRα, is shown in Fig. 5*O*.

##### The Control of DRα Carbohydrate Maturation by DPβ Amino Acid Residues Is Mediated by Interaction with Ii Residues

**FIGURE 6.**
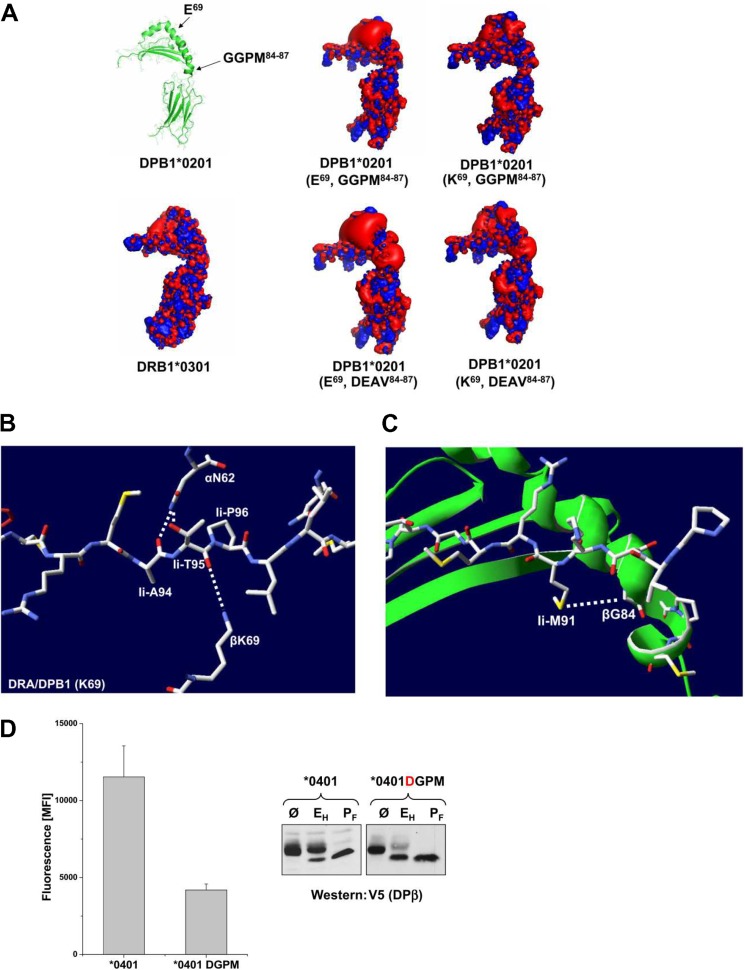
**Interaction of DPβ and DRα subunits with Ii residues.** *A*, charge distribution on DPβ and DRβ subunits. A schematic model of DPB1*0201 the shows positions of Glu-69 and GGPM-(84–87) residues (*upper left*). Visualization of the electrostatic potential of DPβ variants and of DRB1*0301 was performed using the PyMOL program (see “Experimental Procedures”). *B*, interaction of Lys-69 from DPβ to Ii Thr-95 and of Ii Thr-95 to DRα residue Asn-62. The side chain of the Ii Thr-95 adjacent Ii Ala-94 is accommodated in the fourth pocket of the class II heterodimer. *C*, Ii Met-91 binds with its side chain into the first pocket of the class II groove. The Gly-84 residue of DPβ chain does not affect binding of Ii M91 to the class II pocket 1. *D*, mutation of Gly-84 to Asp of the GGPM motif of DPβ. IMRS cells were transfected with DRα and DPβ or DPβ DGPM mutant encoding cDNAs with and without Ii. *Right panel*, cells were lysed, and lysates were left untreated (Ø), digested with Endo H (*E_H_*), or treated with PNGase F (*P_F_*), SDS-PAGE-separated, and Western-blotted for DRβ with V5 mAb. Ii was immunoblotted by mAb Bu43. Surface staining of transfected cells was conducted with mAb 2.06. Mean fluorescence intensity (*MFI*) is displayed in the *left* panel.

So far we have identified two polymorphic sequences of DPβ, namely GGPM-(84–87) and Lys-69, that are important for carbohydrate trimming of the DRα glycoprotein and for cell surface expression of the DRαDPβ heterodimer. By modeling mutated DPB1*0201, we compared the electrostatic potentials of three-dimensional structures containing each of Glu-69/GGPM-(84–87), Lys-69/GGPM-(84–87), Glu-69/DEAV-84–87, and Lys-69/DEAV84–87 together with DRB1*0301 (Fig. 6*A*). The localization of residues 69 and 84–87 in DPβ is shown on the *left image in a ribbon structure*. Comparing the electrostatic potential of DPB1*0201 (Glu-69/GGPM-(84–87)) to the Lys-69/GGPM-(84–87) mutant reveals a dramatic difference of the negative potential in the region that faces the α-chain (Fig. 6*A*, *upper panel*). Comparing the E69K DPB1*0201 mutant with DRB1*0301 (Fig. 6*A*, *upper right panel*) shows that both structures exhibit a more evenly distributed potential. The Lys-69, DEAV84–87 DPβ mutant shows a negative potential adjacent to the location of the first pocket of the peptide binding groove. The strongest negative potential is demonstrated in the Glu-69, DEAV mutant. In the following we searched for interaction of the DPB1*0201 Lys-69 mutant with Ii chain residues based on the assumption that the structure of the DRA.DPB1*0201 Lys-69 mutant resembles the structure of DR3/CLIP (16). We suggest that DPB1*0201 Lys-69 mutant interacts with the polar imide carbonyl group of the peptide backbone of Thr-95 of CLIP (Fig. 6*B*). Because of this interaction, the movement of Thr-95 is restricted, which strengthens the interaction of the Thr-95 side chain with Asn-62 of the α helix of DRα. In contrast, the negatively charged Glu-69 residue of DPβ may repel the polar carbonyl of the peptide bond of Ii Thr-95 and rotate it toward the α helix of DRα and thereby impede the interaction of the Ii Thr-95 side chain with Asn-62 of DRα. Additionally, this conformational impact of Glu-69 from DPB1*0201 on Ii Thr-95 could alter the conformation of the adjacent Ii Ala-94 and impair binding of the methyl group of Ala-94 to the shallow pocket 4 of the MHCII groove.

Comparison of GGPM-(84–87) with the allelic DEAV84-87 sequence of DPβ again reveals negatively charged residues that could affect DPβ interaction with Ii (Fig. 6*A*). Fig. 6*C* demonstrates that Gly-84 has no impact on binding of Ii residue Met-91 (Fig. 6*C*). The orientation of Ii Met-91 in the first MHCII pocket may influence binding of Ii to DRα. Again, the electrostatic potential of the GGPM region resembles the potential of DRB1*0301 and shows, in contrast to the allelic DEAV sequence, no negative potential toward Ii Met-91. The Asp-84 residue of DPβ, however, could impair binding of the hydrophobic side chain of Ii Met-91 to this site. To demonstrate the impact of Asp-84 on DRαDPβ heterodimer formation, we mutated Gly-84 from DPB1*0401 to Asp-84. The mutated DPβ chain was co-expressed with DRα and Ii. Endo H treatment of the mutated DPβ chain revealed that most of the β chains remained Endo H-sensitive (Fig. 6*D*, *right panel*). For comparison, Endo H-resistant DPB1*0401 is shown. The DRα chain in combination with DPB1*0401 DGPM exhibited partial Endo H resistance for DRα (not shown). Cell surface expression of DRA.DPB1*0401 DGPM was strongly reduced compared with DPB1*0401 (Fig. 6*D*, *left panel*). This result demonstrates the influence of Asp-84 on the formation of the DRαDPβ heterodimer.

Neither Lys-69 nor GGPM-(84–87) of DPβ alone yielded effective glycosylation of DRα and surface expression of DRαDPβ. Thus, a cooperative effect of the polymorphism encompassing residues 69 and 84–87 controls assembly of DPβ with DRα. The pairing of DRα and DPβ depends on interaction of Ii with DPβ residues that stabilize the conformation of the DRαDPβ heterodimer. We conclude that two negatively charged regions on DPβ influence interaction with Ii residues. A reorganized orientation of Ii residues may yield a long-distance impact on the DRα conformation. This conformational change may explain the aberrant glycosylation of the DRα carbohydrate and impaired surface expression of the isotype mismatched MHCII heterodimer.

##### Origin of the Lys-69/GGPM-(84–87) Sequence

**FIGURE 7.**
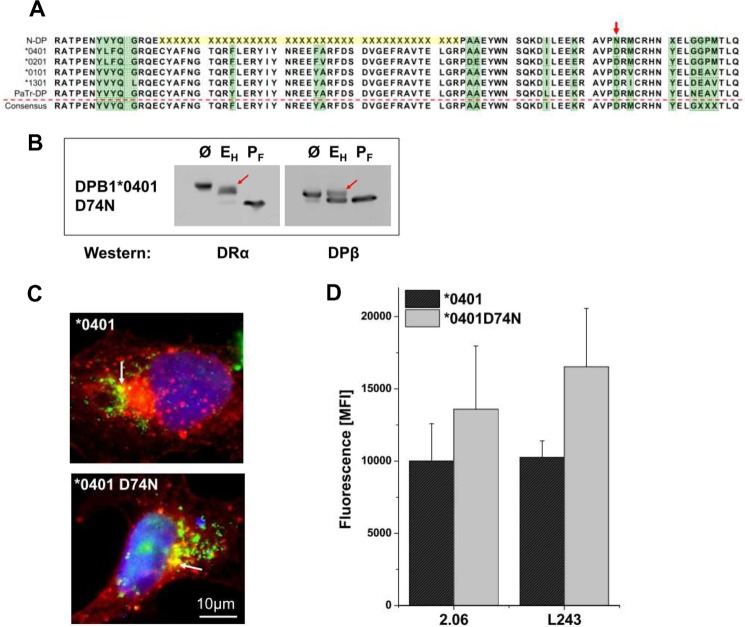
The Neanderthal DPβ sequence is related to the HLA DPB1*0401 allele. *A*, alignment of the first 90 aa residues (β1 domain) of DPB1* from Neanderthal (*N-DP*), with human alleles 0401, 0201, 0101, and 1301 and from chimpanzee (*PaTr*) sequences. The β2 domain sequence shows limited variation and, therefore, is not shown. Polymorphic residues are in *green*, and Neanderthal residues not available are indicated by *X*. A species-specific aa residue is indicated by an *arrow. B*, the DPB1*0401 sequence was mutated in Asp-74 to Asn. IMRS cells transfected with the mutated DPβ chain, with DRα, and with Ii were lysed and digested with Endo H (*E_H_*), with PNGase F (*P_F_*), or left untreated (Ø) and subsequently blotted for DRα (*left*) or for DPβ (*right panel*). *C*, transfected cells (DRA, Ii and DPB1*0401 or DB1*0401 D74N) were stained for CD63 (*green*) and for class II (*red*). *Arrows* indicate vesicles with colocalization of DRαDPβ with CD63. *D*, cells were transfected as in *B*, and fluorescence intensity was compared with DRA.DPB1*0401 transfected cells. Cells were stained with class II mAbs 2.06 and L243.

To explore the evolutionary origin of the Lys-69/GGPM-(84–87) sequence we inspected sequences of primates and compared the DPB1*0401 sequence and other human alleles to genomic sequences available from the Neanderthal genome. The Neanderthal sequence was obtained from three individuals and, therefore, does not represent a unique allelic sequence. Fig. 7*A* demonstrates that Lys-69 and GGPM-(84–87) is present in the Neanderthal genome. The GGPM motif was not found in the DPβ sequence of chimpanzees or in any other examined primate sequence (not shown). The Neanderthal DPβ sequence is similar to that of the human DPB1*0401 allele. In the Neanderthal β1 sequence only one residue (Asn-74) is not found in human alleles. The available Neanderthal sequence did not resolve aa residues 25–53 and residue 81. However, this sequence is highly conserved between human and chimpanzee leaving only Tyr-24 *versus* Phe as a difference. The comparison suggests that this sequence is also conserved in the Neanderthal genome. The amino acid residues VY at positions 8 and 9 in the Neanderthal sequence, compared with LF in the 0401 allele, do not obstruct formation of the DRDP heterodimer (compare polymorphic patch 1 in Fig. 5*O*). To examine whether a species-specific sequence impacts on heterodimer formation with DRα, the aa residue Asn-74 of the Neanderthal sequence was introduced into the DPB1* 0401 allele. The mutated DPβ chain was co-expressed with DRα and Ii. Subsequently, carbohydrate maturation (Fig. 7*B*), intracellular localization (Fig. 7*C*), and cell surface expression (Fig. 7*D*) was assessed. Endo H and PNGase F digest of cell lysates (Fig. 7*B*) revealed that DRα and mutated DPβ chain displayed normal glycosylation, with partial Endo H-resistant DRα (*left panel*) and complete Endo H-resistant DPβ band (*right panel*). To examine intracellular localization of DRαDPβ, cells were grown on coverslips and stained with CD63 mAb and with LGII-612.14 (LGII) mAb against class II. Costaining of LGII with the late endosomal marker CD63 (*arrows*) indicates that the DRA.DPB1*0401 D74N heterodimer was localized to the class II peptide loading compartment. Surface staining of DRA.DPB1*0401 Asn-74 was compared with DRA.DPB1*0401. For staining, cells were incubated with two mAbs. mAb L243 detects peptide loaded class II molecules on the cell surface, whereas mAb 2.06 in addition reacts with empty α-β heterodimers and, therefore, detects all surface-exposed class II molecules. Surface detection of D74N-mutated DPB1*401 in Fig. 7*D* demonstrates that the DRαDPβ receptor is capable of presenting peptides at the cell surface.

**FIGURE 8.**
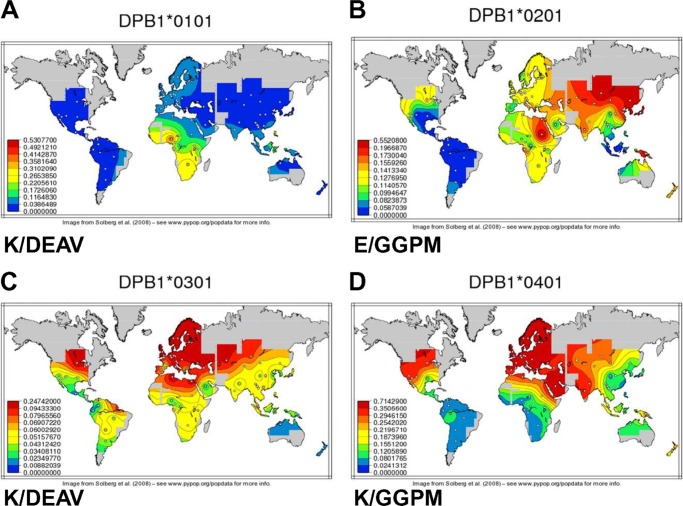
**World-wide distribution of DPB alleles and the Lys/GGPM motifs.** The maps display the allele frequencies of the DPB alleles 0101, 0201, 0301, and 0401 on the basis of non-migratory populations. Higher frequencies are displayed in *red*, and lower frequencies are *blue*. The frequency scaling between the maps is not identical and is displayed as *inset*. The DPB1*0401 allele (*panel D*) is relatively rare in sub-Saharan Africa but progressively more frequent in northern European populations (*red*). Neanderthals carry both Lys-69 and GGPM-(84–87) and differ from DPB1 from modern human by a single residue over the available sequence. Note though that Lys-69 and GGPM-(84–87) occur independently in other alleles, which makes interpretation of the origin of their arrangement difficult to determine.

To search for the provenance of the Lys-69/GGPM-(84–87) combination, we examined the distribution of four DPB alleles worldwide. The maps on Fig. 8 show the gradients of the first four DPB1 alleles (38, 39). Both Lys-69 and the GGPM motif are distributed over the African continent at a reasonable frequency. The combination of Lys/GGPM reaches moderate to high levels in North Africa and is particularly prevalent in Scandinavia. Note also the low levels of Lys/GGPM in South America. The fact that the Neanderthal DPB* allele resembled 0401 may indicate that Lys/GGPM was acquired by introgression from these ancient populations. However, DPB alleles are prone to recombine dimorphisms distributed throughout their sequences, and the Lys and GGPM motifs may have had their origin purely within *H. sapiens*. In either case it is possible that formation of an allelic product of DPB that could heterodimerize with DRA was subject to selection. Whether this selection was positive, particularly over the northern hemisphere, or disadvantageous over areas of Africa and South America remains uncertain.

## DISCUSSION

The αβ heterodimer composed of DRα and DPB1*0401 is an example of a functional isotype-mixed αβ heterodimer. To unravel the impact of polymorphic residues influencing class II α and β subunit assembly, we systematically determined the interaction of polymorphic residues in DPβ allotypes with DRα. These experiments were conducted in the presence or absence of the chaperone Ii. Ii controls carbohydrate maturation of the α-β heterodimer, and we show here that the DRA.DPB1*0401 molecules acquire SDS resistance (indicating peptide binding) and are expressed on the cell membrane. We demonstrate that allotypic differences in DPβ determine the assembly of isotype-mixed DRαDPβ heterodimers with Ii. Three additional DPβ allotypes associate with the DRα glycoprotein in the presence of Ii and form complexes, but their conformations did not lead to appropriate carbohydrate maturation. One complex composed of DRα and DPB1*0201 was exported from the ER and reached the cell surface, but even in the presence of Ii it did not acquire the conformation required for a normal carbohydrate modification. Two further isotype-mixed αβ combinations, which were co-expressed with Ii, formed complexes containing Ii but did not assemble as heterodimers. An imperfect conformation of αβ heterodimers yields aggregation rather than a rescue of conformation preceding binding of peptides (40). Lack of surface transport has been ascribed to a mechanism for limiting the expression of inappropriate paired murine MHCII subunits (41).

Ii promotes optimal MHCII assembly that results after release of Ii in a receptor susceptible for peptide binding. When the structure of different peptide-MHCII complexes was compared, the conformations of side chains of MHCII residues contacting the peptide were highly conserved (18). Possibly, Ii converts the αβ heterodimer into a unique conformation that is sustained after binding of the peptide. Consistently, various peptides confer only minimal rearrangement of MHCII residues (18). Upon release of the Ii-derived fragment CLIP from the MHCII cleft, the heterodimer becomes more flexible. This was previously demonstrated by a molecular dynamic simulation study (42).

The groove binding segment of Ii (CLIP) appears to be critical to shape the conformation of the MHCII peptide receptor. In recent years recombinant Ii chains have been described in which the CLIP sequence was replaced by the sequence of antigenic peptides (43, 44). During assembly of α and β subunits in the ER, the Ii fusion protein is embedded in the MHCII cleft, and the MHCII molecule presents the antigenic peptide on the cell surface for activation of CD4^+^ T cells. One can envisage that some Ii-antigen fusion proteins may select unusual isotype-mismatched MHCII heterodimers to assemble on the scaffold of the antigenic peptide in the ER.

The structural requirements for isotype-mismatched MHCII subunit assembly have been explored by species mixed combinations of DRα with mouse IA^k^ β chain and monitoring of cell surface expression (45). Construction of chimeric molecules of two HLA isotypes or interspecies fusion proteins indicate that the β1 domain impacts on isotype matched assembly of MHCII heterodimers (46). Cell surface expression of Aα^k^Aβ^d^ heterodimers was induced by mutation of polymorphic residues at the position 76 and 86 of Aβ^k^ chain (47). Interaction of Ii with the MHCII groove is an essential requirement for assembly of some α and β allotypes. Amino acid residues from both the α and the β chains form the P1 pocket. This pocket has an important role for interaction of the MHCII subunits, for contact of the upper-lower domains of DR1, and for binding of Ii (35). In DR1, P1 shows a preference for binding of aromatic side chains and aromatic dipeptides can modulate peptide binding (48). Simulation of a mutation in P1 revealed a long range control of conformation of the complete MHCII binding cleft (42). Partial filling of the P1 pocket by a mutation of β Gly-86 to Tyr stabilizes the compact form of the empty DR1 dimer (35, 49). Mutation of Ii Met-91 to Gly, the anchor residue for binding to P1, abolishes co-isolation of Ii with a single DRα (27). In the experiment presented here the Ii M91G mutant yielded impaired trimming of the DRα glycan and reduced surface exposure of DRα co-expressed with DPB1*0401 and Ii. DPβ residues that are involved in formation of P1 form a polymorphic cluster. It appears that mutations of DPβ residues of the P1 pocket lead to a conformational change that results in altered carbohydrate trimming of the DRα glycoprotein. Binding of Ii to DRαDPβ Lys-69/GGPM-(84–87) allotypes results in a structure that supports trimming of the DRα carbohydrate. We show here that the P1 pocket plays an important role for interaction of α, β, and Ii residues in the isotype-mixed MHCII heterodimer.

Some DPβ allotypes with Glu-69 are involved in development of chronic beryllium disease, an inflammatory T cell-mediated disease. DPβ molecules with Lys-69 cannot bind beryllium and, therefore, elicit no T cell response. The polymorphism in position 69 of DPβ was shown to impact on antibody epitopes, suggesting a conformational change of the DP molecules (51). In addition, T cell recognition is influenced by residues 69 and 84–87 of DPβ (52). The locations of the anchor positions P1 and P4 are conserved, and the polymorphic residues 69 and 84–87 determine allele-specific preferences for anchor positions. The published data together with our data suggest that the residues in position 69 and 84–87 of DPβ may have a general function in assembly of class II heterodimers.

Expression of isotype-mixed class II peptide receptors could broaden the repertoire of a wider array of antigens presented by an individual antigen presenting cell. The structural constraints of DRαDPβ assembly presented in this paper could in addition point to an evolutionary process in generating allelic diversity within a subfamily of class II peptide receptors. Recently, it was discovered that some HLA class I alleles in the European population were from ancient origin and presumably transmitted by resident Neanderthals to immigrating humans (53). It is assumed that the resident Neanderthals had immune systems adapted to local pathogens in Europe. We found that the Lys-69/GGPM-(84–87) motif, which is essential for formation of DPβ to a heterodimer with DRα, is present in both Neanderthals and modern humans. The DPB1*0401 allele, which contains the Lys/GGPM motif, shows a phenotypic frequency of only 11% in sub-Saharan Africa, whereas 68% of the European population carry this allele (50). The compatibility of the Neanderthal DPβ sequence in DPβDRα heterodimers suggests that a Neanderthal gene was introduced into the *H. sapiens* genome by admixture of the two human species. This HLA class II peptide receptor may have evolved further in ancient *H. sapiens* to a novel DPβ family in modern humans. If this interpretation is substantiated it may indicate that the introgression of the Neanderthal DPB allele provided a selective advantage. The nature of any such advantage is difficult to determine, but as we have shown, it may relate to the formation of novel DRADPB-mixed isotype molecules.

## Footnotes

3 The abbreviations used are:

ER: endoplasmic reticulum
aa: amino acid
Endo H: endoglycosidase H
PNGase F: peptide *N*-glycosidase F
MHCII: major histocompatibility complex class II
Ii: invariant chain
HLA: human leukocyte antigen
contig: group of overlapping clone.
